# Six ans de survie sans progression après chimiothérapie pour une tumeur neuro-ectodermique périphérique rénale métastatique

**DOI:** 10.11604/pamj.2019.32.42.12475

**Published:** 2019-01-23

**Authors:** Ali Sbai, Zineb Dahbi, Farid Naciri, Fouad Elmejjatti, Amine Guerouaz, Kawtar Lakhmis, Mohammed Guezzar, Fadwa Allouch, Loubna Mezouar

**Affiliations:** 1 Service de Radiothérapie, Centre Hospitalier Universitaire Oujda, Maroc

**Keywords:** Tumeurs neuro-ectodermiques primitives, PNET rénale, chimiothérapie, Primary neuroectodermal tumor, PNET of the kidney, chemotherapy

## Abstract

Les tumeurs neuro-ectodermiques périphériques (PNET) rénales sont extrêmement rares, souvent diagnostiquées à un stade tardif compte tenu de leurs présentations cliniques aspécifiques. Le traitement des stades métastatiques repose sur la chimiothérapie palliative, nous rapportons un cas de PNET rénale métastatique d’emblée au niveau ganglionnaire et cutané, qui a présenté une bonne réponse clinique et radiologique et sans signe de progression depuis 6 ans après la fin d’une polychimiothérapie par vincristine, doxorubicine et cyclophosphamide.

## Abstract (en)

Peripheral neuroectodermal tumors (PNET) of the kidney are extremely rare. They are often diagnosed at a late stage due to their nonspecific clinical presentation. Treatment of patients with metastases is based on palliative chemotherapy. We here report a case of PNET of the kidney with sudden onset of metastases to the lymph nodes and to the skin. The patient showed good clinical and radiological response and experienced progression-free survival at 6 years after polychemotherapy with vincristine, doxorubicin and cyclophosphamide.

## Introduction

Les PNET rénales sont des tumeurs malignes exceptionnelles, et hautement agressives, qui se voient essentiellement chez l’adulte jeune, elles se manifestent cliniquement par des signes urinaires aux stades localement avancés et métastatiques, ce qui explique leur diagnostic tardif et leur pronostic fâcheux. Nous rapportons ici un cas clinique de PNET rénale métastatique ayant bien évolué sous chimiothérapie.

## Patient et observation

**Figure 1:**
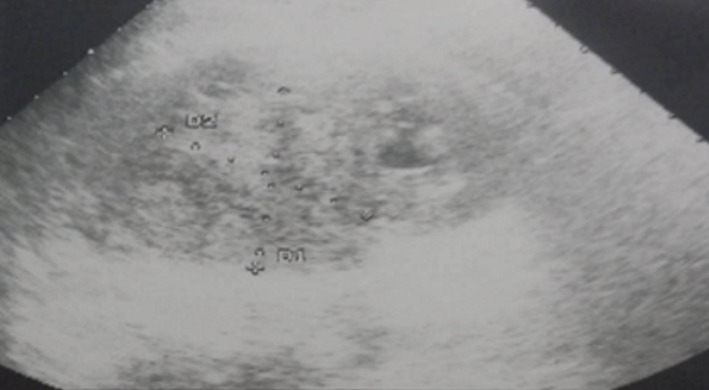
Aspect échographique de la masse rénale gauche

**Figure 2:**
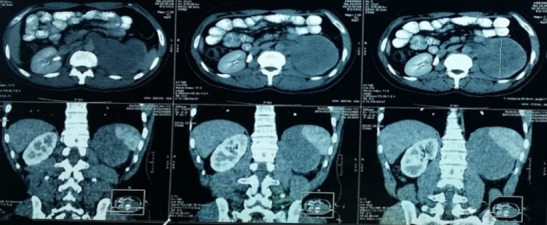
Aspect de la TDM abdomino-pelvienne initiale objectivant une énorme masse rénale gauche

Il s’agit de M. B.M, âgé de 30 ans, maçon de profession, un tabagique chronique à raison de 30 paquets années, sans antécédents pathologiques particuliers, qui nous a été adressé pour la prise en charge de lombalgies chroniques associées à une hématurie totale évoluant dans un contexte d’apyrexie, d’amaigrissement non chiffré et de fléchissement de l’état général. L’examen clinique a trouvé un patient en mauvais état général (PS = 2) qui présente un contact lombaire gauche, une masse para-lombaire droite avec des lésions cutanées sur la face inféropostérieure de la cuisse droite et du creux poplité droit. L’échographie pelvienne a objectivé une masse médio rénale de 54mm sans dilatation pyélocalicielle (Figure 1); la TDM thoraco-abdomino-pelvienne a montré une formation tissulaire d’allure tumorale qui occupe tout le rein gauche de 10cm de grand axe, englobant l’artère rénale gauche, la surrénale et le muscle psoas homolatéraux en bas, et se développant en haut vers l’aorte abdominale et la rate sans les envahir, sans signes de thrombose de la veine cave inférieure, et sans autres lésions pulmonaires, hépatiques ni osseuses (Figure 2).

Une biopsie exérèse de la masse para lombaire droite a été réalisée, dont l’étude anatomopathologique est revenue en faveur d’une localisation métastatique sous cutanée d’une tumeur neuroectodemrique périphérique, avec une prolifération tumorale dermique et hypodermique à cellules rondes, organisées en nappes et en cordons anastomosés, les cellules tumorales étaient de taille moyenne, à cytoplasme réduit et à noyau discrètement anisocaryotique; la chromatine était finement granulée et il s’y associait de nombreuses figures de mitoses. Le complément par étude immuno-histochimique a montré un marquage positif des cellules tumorales avec les anticorps anti PS100, anti CD99, anti chromogranine et anti synasptophysine, et l’absence de marquage aux anticorps anti-AE1,antiAE3, antiEMA, anti actine, anti desmine et anti melanine A, confirmant le diagnostic de PNET rénale métastatique au niveau cutané. Sur ceci nous avons classé la tumeur T4N1M1 selon la classification TNM2009, le patient a bénéficié d’une polychimiothérapie palliative à base de cyclophosphamide 1.2g/m2 J1, doxorubicine 75mg/m2 J1 et vincristine 1.5mg/m2 J1, J1 = J21. Nous avons constaté une amélioration très notable de son état général après la 1^ère^ cure et une disparition quasi complète de toutes les lésions cutanées pré existantes après les 3 premières cures, vue cette excellente réponse clinique et radiologique, nous avons convenu de continuer jusqu’à 9 cures, avec des délais inter-cures respectés et une excellente tolérance clinique et biologique.

**Figure 3:**
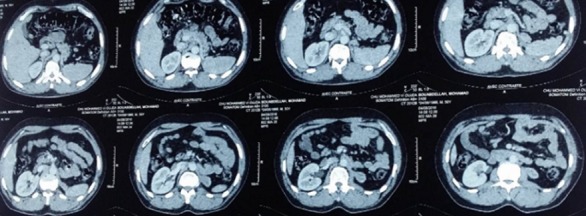
Aspect de la TDM Thoraco-abdominopelvienne post-thérapeutique montrant la nette régression du processus tumoral rénale gauche

L’évaluation clinique, après 9 cures de chimiothérapie, trouve un patient en excellent état général, une disparition complète de toutes les lésions cutanées, sans contact lombaire, sur le plan radiologique: à la TDM thoraco-abdomino-pelvienne, nous notons une nette régression de la masse de la loge rénale gauche, faisant 4x2x1.5cm versus 10cm de grand axe, sans apparition de nouvelles lésions. Le patient a fait l’objet d’une surveillance clinique et radiologique régulière, à un rythme trimestriel les 2 premières années, puis semestrielle jusqu’aujourd’hui. Actuellement, en décembre 2016, soit 78 mois de la fin du traitement, il se porte bien, sa réinsertion professionnelle est complète, son examen clinique ainsi que sa dernière imagerie par TDM thoraco-abdomino pelvienne sont en faveur d’un bon contrôle locorégional et à distance de sa maladie (Figure 3).

## Discussion

Les tumeurs neuro-ectodermiques primitives rénales ont été décrites pour la première fois par STOUT en 1918, connues dans la littérature sous différentes dénominations: tumeur neuro-ectodermique primitive, neuroépithéliome malin, neuroblastome périphérique, tumeur neuroectodermique primitive des tissus mous ou sarcome neuroectodermique périphérique des tissus mous [1]. Elles constituent un sous-groupe des tumeurs neuroectodermiques primitives malignes qui représentent 1,1% de l’ensemble des cancers de la population générale, dont la majorité se développent au niveau de l’os et des tissus mous (sarcome d’Ewing) [2], avec une prédilection (40%) pour la région thoraco-pulmonaire appelée tumeur d’Askin et les extrémités (25%). Les PNET rénales constituent la localisation viscérale primitive la plus fréquente: peu de cas sont rapportés dans la littérature, les auteurs se sont mis d’accord sur le potentiel agressif de ces tumeurs, qui métastasent habituellement au niveau ganglionnaire, hépatique, rénal et osseux.

L’âge moyen de survenue des PNET rénales est de 32,7 ans (7 à 62ans). Il n’existe pas de prédominance de sexe. Des antécédents personnels de lymphomes malins hodgkiniens ou non hodgkiniens traités par chimiothérapie et/ou radiothérapie, de SIDA, de neurofibromatose ont été décrits. Notre patient âgé de 30ans, n’avait pas d’antécédents de ce type [3]. L’expression clinique des PNET rénales n’est pas pathognomonique. Le délai diagnostique est relativement court, témoignant de son agressivité. Les signes urinaires de type lombalgies, coliques néphrétiques, masse palpable sont leurs principales manifestations, l’hématurie et l’altération de l’état général traduisent un stade évolué de la maladie et signent un mauvais pronostic. Le bilan radiologique doit comporter une imagerie par tomodensitométrie qui montre souvent un syndrome de masse avec des zones de nécrose ou d’hémorragie. En IRM, la tumeur est en hyper signal dans les séquences T1 et T2 [4]. Notre patient a bénéficié d’une TDM thoraco-abdominopelvienne qui a montré une formation tissulaire d’allure tumorale, de 10cm de grand axe, au dépends du rein gauche, englobant l’artère rénale homolatérale, la surrénale gauche, le muscle psoas et l’aorte abdominale, sans signes de thrombose de la veine cave inférieure. Sans autres lésions à distance. L’IRM abdomino-pelvienne n’a pas été réalisée chez notre patient.

Biologiquement, Les catécholamines sanguines et leurs métabolites urinaires peuvent être élevées mais à des concentrations moindres qu’au cours des neuroblastomes. En cas de volumineuse tumeur rénale le dosage pré et post-opératoire de la Neuron Specific Enolase (NSE) sérique permet un suivi post thérapeutique [5]. Notre patient n’a pas bénéficié d’un dosage de NSE sérique vue leur indisponibilité au sein de notre structure. Le diagnostic de PNET rénale repose sur l’examen anatomopathologique. Macroscopiquement la tumeur est arrondie, ovalaire ou multi nodulaire, bien limitée, sans encapsulation. A la coupe, elle est gris-beige ou jaune et sa consistance est molle ou friable. Les remaniements nécrotiques ou hémorragiques sont fréquents. Des calcifications sont parfois observées [6]. L’histologie retrouve une prolifération maligne, d’architecture lobulaire, pseudo-alvéolaire, constituée de petites cellules rondes ou ovalaires, d’allure monomorphe et peu différenciée. L’anticorps anti-Neuron Specific Enolase (NSE) est un marqueur neural non spécifique mais qui s’exprime, sous la forme d’un marquage cytoplasmique net et diffus, dans 90 à 95% des MPNT y compris dans les localisations rénales. L’anticorps dirigé contre la glycoprotéine membranaire p30/32 MIC2 est exprimé dans 84 à 100% des tumeurs neuroectodermiques primitives et plus de 95% des sarcomes d’Ewing. Sa spécificité est donc relative. D’autres marqueurs (neurofilament triplet protéines, béta-tubuline et protéines associées aux microtubules, synaptophysine, protéine S100, chromogranine A, Leu-7, béta-2-microglobuline, vimentine) sont exprimés de façon variable. La cytogénétique permet de caractériser les MPNT grâce à une anomalie spécifique, la translocation t(11;22)(q24;q12), qu’elle soit simple, complexe ou variante. La compréhension de cette anomalie chromosomique permettra peut-être, dans l’avenir la mise au point de traitements spécifiques. Notre patient a présenté une prolifération tumorale dermique et hypodermique à cellules rondes, organisée en nappes et en cordons anastomosés, les cellules tumorales étaient de taille moyenne, à cytoplasme réduit et à noyau discrètement anisocaryotique; la chromatine était finement granulée et il s’y associait de nombreuses figures de mitoses. Le complément par étude immuno-histochimique a montré un marquage positif des cellules tumorales avec les anticorps anti PS100,anti CD99, anti chromogranine et anti synasptophysine, et l’absence de marquage aux anticorps anti-AE1,antiAE3, antiEMA, anti actine, anti desmine et anti melanine A, confirmant le diagnostic d’un PNET rénal métastatique au niveau cutané. Pour les stades localisés, le traitement principal des PNET repose sur la chirurgie d’exérèse, avec des marges minimales de 1cm de tissu sain [7]. Le recours aux traitements adjuvants est une question en cours d’évaluation [8, 9]. Sur la plus large série rétrospective des PNET rénales publiée par Thivally en 2008, regroupant un total de 16 patients, ayant tous bénéficié d’une chimiothérapie adjuvante, la radiothérapie a été proposée pour les patients ayant eu une exérèse chirurgicale incomplète, d’une dose de 50 à 60Gy. Une réponse complète a été obtenue pour l’ensemble des patients de la série qui présentait une maladie localisée [10]. La survie globale des patients avec une maladie localisée, après la chirurgie d’exérèse complète est de 60 mois, comparé à 15mois pour les patients avec une maladie résiduelle au stade non métastatique, et ceci malgré le traitement adjuvant, ce qui consolide la place de la chirurgie comme l’arme thérapeutique majeure dans le traitement des PNET rénales localisées.

Le diagnostic souvent tardif de ces tumeurs explique la fréquence des cas localement avancés, où la chirurgie carcinologique ne peut se concevoir, et le recours aux autres moyens thérapeutiques trouve toute sa place, notamment: la chimiothérapie qui permet d’optimiser le traitement local et de contrôler la maladie métastatique via son action systémique. Les drogues cytotoxiques actives sur les PNET rénales sont multiples, souvent utilisées en association pour un meilleur résultat thérapeutique, en absence de standards thérapeutiques [10]. Les protocoles décrits sur les séries publiées sont variables; la vincristine, l’adriyamycine et le cycolphosphamide sont les drogues cytotoxiques les plus utilisées. L’étoposide et l’ifosfamide semblent également avoir de bons résultats [11]. Vu leur rareté et la similitude biomoléculaire entre les sarcomes d’Ewing et les PNET, l’usage d’une chimiothérapie similaire paraît plausible, d’ailleurs les essais cliniques randomisés en quête du meilleur protocole de chimiothérapie à proposer pour ces situations cliniques incluent à la fois les PNET et les sarcomes d’Ewing [12]. L’essai Sarcoma III suggère l’intérêt de l’introduction des molécules étoposide et ifosfamide en alternance avec le protocole VAC-doxorubicine avec un bénéfice en survie sans récidive à 3 ans (69% versus 50%) et un bénéfice en survie globale à 3ans (80% versus 56%) [13, 14].

Notre patient a bénéficié de 9 cures d’une polychimiothérapie palliative à base de cyclophosphamide 1.2g /m2 J1, doxorubicine 75mg/m2 J1 et vincristine 1.5mg/m2J1, J1 = J21, avec une excellente tolérance clinique et biologique. Le pronostic des PNET reste mauvais. La médiane de survie sans lésion tumorale après traitement est de 50% à 3 ans et de 35 à 45% à 5 ans. Le pronostic dépend essentiellement de l’existence de métastases, du volume tumoral et du traitement reçu, le traitement chirurgical associé à une chimiothérapie s’accompagnant du meilleur pronostic. Le délai moyen de survie est de 12 mois pour les stades métastatiques [15, 16]. Pour notre patient, la chimiothérapie de type VAC a permis un contrôle locorégional et à distance de la maladie, sans signe de progression depuis 78 mois de la fin du traitement.

## Conclusion

Vu l’immense difficulté de proposer un protocole thérapeutique de référence pour les tumeurs neuro-ectodermiques rénales métastatiques, et compte tenu de leur rareté, nous rapportant un cas clinique d’un succès thérapeutique grâce à une polychimiothérapie associant le cyclophosphamide, la doxorubicine et la vincristine.

## Conflits d’intérêts

Les auteurs ne déclarent aucun conflit d'intérêts.
